# HIV, immune activation and salt-sensitive hypertension (HISH): a research proposal

**DOI:** 10.1186/s13104-019-4470-2

**Published:** 2019-07-16

**Authors:** Sepiso K. Masenga, Benson M. Hamooya, Selestine Nzala, Geoffrey Kwenda, Douglas C. Heimburger, Wilbroad Mutale, John R. Koethe, Annet Kirabo, Sody M. Munsaka

**Affiliations:** 1 School of Medicine and Health Sciences, Mulungushi University, Livingstone, Zambia; 2 Department of Biomedical Sciences, School of Health Sciences, University of Zambia, Lusaka, Zambia; 3 Vanderbilt Institute for Global Health, Nashville, TN USA; 4 Department of Epidemiology and Biostatistics, School of Public Health, University of Zambia, Lusaka, Zambia; 5 Department of Medical Education Development, University of Zambia, Lusaka, Zambia; 6 Department of Health Policy and Management, School of Public Health, University of Zambia, Lusaka, Zambia; 7 Division of Infectious Diseases, Vanderbilt University Medical Center, Nashville, TN USA; 8 Division of Clinical Pharmacology, Vanderbilt University Medical Center, Nashville, TN USA; 9 Department of Molecular Physiology and Biophysics, Vanderbilt University, Nashville, TN USA

**Keywords:** Hypertension, Immune activation, Salt-sensitivity, HIV

## Abstract

**Objective:**

The objective of this study is to quantify and compare the effect of excess dietary salt on immune cell activation and blood pressure in HIV versus HIV negative individuals.

**Results:**

Salt-sensitivity is associated with increased immune cell activation in animal studies. This concept has not been tested in people living with HIV. This study will therefore add more information in elucidating the interaction between HIV infection and/or anti-retroviral therapy (ART), immune-activation/inflammation and hypertension.

**Electronic supplementary material:**

The online version of this article (10.1186/s13104-019-4470-2) contains supplementary material, which is available to authorized users.

## Introduction

Salt-sensitive hypertension is defined as a change in blood pressure (BP) greater than 10% or > 5 mmHg in response to either increased or reduced salt intake [1, 2]. This phenomenon is more common in individuals with higher BP, blacks, elderly, and is seen in certain comorbid conditions such as chronic kidney disease, diabetes mellitus or metabolic syndrome [3]. Salt has been implicated to initiate an inflammatory process that could result in hypertension [4–6]. Moreover, inflammation-driven salt accumulation is evident on sites of infection and/or inflammation [7].

Antiretroviral (ART) treated People living with HIV (PLWH) are prone to development of hypertension and elevated BP [8–10]. One of the reasons for this could be the interaction of a generalized systemic suboptimal immune activation and inflammation that persists, traditional risk factors including dietary salt intake, HIV particles and ART [11, 12]. However, the effect of salt on BP and immune activation has not been tested in PLWH and dietary assessments are never part of routine assessment in the clinical setting especially in Africa hence the need for this proposed study.

Study objectives:

1. To determine the prevalence of salt-sensitive hypertension in PLWH and compare with HIV negative population
2. To quantify and compare the effect of excess dietary salt on immune cell activation and BP in ART-treated normotensive, ART-treated hypertensive, HIV negative hypertensive and HIV negative normotensive individuals.

## Main text

### Methods

#### Study site, design and patients

We propose to conduct a three-week prospective study at Livingstone Central Hospital (LCH) involving PLWH attending routine ART and HIV negative controls from volunteer health workers who will be matched for age, sex, body mass index (BMI), and waist circumference (WC). LCH is the largest referral hospital in Southern Province of Zambia that offers ART and general medical services to the community with approximately 3776 PLWH enrolled in ART.

#### Eligibility criteria

##### Inclusion criteria

The study cohort will include all adults (aged 18 and above) who will be required to verbally consent and sign a consent form and should be attending medical clinic for both general clinics and ART. Four groups will be established for comparison namely:

1. ART treated normotensive individuals
2. ART treated hypertensive individuals
3. Hypertensive individuals who are HIV negative
4. Health controls.

##### Exclusion criteria

Exclusion from the study will be based on existence of co-morbidities such as diabetes mellitus and cancer, and also those with existing and recent past opportunistic infections, syphilis, hepatitis C and B virus infection and tuberculosis infection; and sick patients (clients seeking healthcare due to an illness rather than routine ART clinic reviews) will be excluded. Those with recent and current alcohol consumption and smoking status will also be excluded from the study.

#### Study variables

##### Response variables

Primary: Salt-sensitivity of BP

Secondary: Markers of antigen presenting cell activation CD80, CD83, CD86, CD69 and IsoLGs, pro-inflammatory markers such as interleukin 17A (IL-17A), IL-6, tumor necrosis factor alpha (TNF-α), interferon gamma (IFN-γ) and intermediate monocyte subset (CD14^++^CD16^+^) which are raised in inflammation.

##### Explanatory variables

Traditional risk factors: age, sex, WC, BMI, waist-to-hip ratio (WHR), sedentary life style, physical activity, fruit and vegetable intake, hours of sleep.

Clinical risk factors: BP, Electrocardiogram (ECG) parameters, lipid profile, Urine and blood electrolyte (sodium, potassium and chloride), Urine macro- and micro-analysis, ART regimen, duration on ART, adherence to ART, family history of hypertension and diabetes risk, duration of hypertension, medical history.

Immune related factors: Immune status (CD4 and HIV RNA viral load absolute counts), soluble CD14 (sCD14), Complete blood count, C-reactive protein (CRP).

#### Sample size

For aim 1, OpenEpi online software (Kelsey’s method) will be used to compute a total sample size of 236 (118 hypertensive versus 118 normotensive in 1:1 ratio each between PLWH and HIV negative groups) for salt-sensitive hypertension with the following inputs: 95% significance level at 80% power; Estimated percent of salt-sensitive hypertension in the hypertensive and normotensive population was 50% and 25% respectively [13–15].

For aim 2, G*Power [16, 17] version 3.1.9.4 will be employed to calculate a total sample size of 48 (12 for each group). The input assumptions are detailed in Table 1.

**Table 1:** Sample size determination

F tests using MANOVA for repeated measures, within–between interaction		
Options	Pillai V, O’Brien-Shieh Algorithm	
Analysis	A priori: Compute sample Size	
Input	Effect size f(v)	1
	α err prob	0.05
	Power (1-β err prob)	0.80
	Number of groups	4
	Number of measurements	3
Output	Non-centrality parameter λ	22
	Critical F	2.8477260
	Numerator df	6.0
	Denominator df	14.0
	Sample size	11 per group
	Lost to follow (10%)	1 per group
	Total sample size	48
	Actual power	0.825
	Pillai V	1.0

#### Sampling

For selection of hypertensive and normotensive study participants, simple random sampling will be used assisted by an online random number generator. PLWH will be randomly selected during their routine medical visits.

In order to create equal groups in the strata, usage of randomization blocked in blocks of size four and six will be employed.

##### Procedure for aim 1

**Fig. 1:**
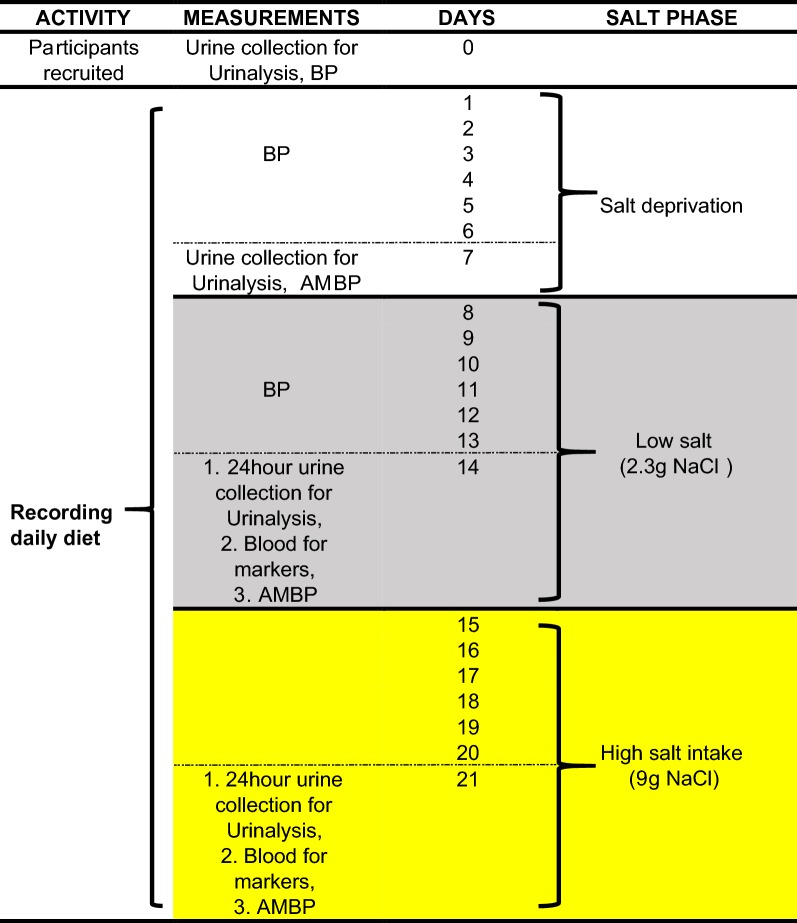
In the salt deprivation phase, Participants will be recruited on day 0, urine sample collected and requested to avoid adding salt to their food or consume processed foods that contain salt for the next 7 days. In the low salt phase, participants will be provided with 2.3 g of sodium everyday apportioned in three parts to add to their meals. In the high salt phase participants will be provided with 9 g of dietary salt and split as previously described above. Blood pressure (BP) will be measured everyday (day 0 to 21) between 17:00 and 19:00 h or between 06:00 and 08:00 h. Ambulatory blood pressure (AMBP) will be measured on days 7, 14 and 21. A 24-h urine will be collected on days 14 and 21 for urinalysis

In order to determine the prevalence of salt-sensitive hypertension in PLWH and HIV negative population, we will conduct salt-sensitive analyses as described below:

*Salt sensitivity analysis* We will employ a modification of the procedure described by He et al. [18] as shown in Fig. 1. Briefly, in the 1st week (7 days), participants will be requested to avoid consuming processed foods and adding salt to their food. Participants will be required to record their daily diet. In the 2nd week, they will be given the World health organisation (WHO) recommended low salt (2.3 g), and in the 3rd week, high salt (9 g/day). The changes for low salt (2.3 g NaCl/day) on BP will be calculated as previously described [18]. BP will be monitored three times each day and on the last day of each phase a 24-h BP using an ambulatory BP monitoring device will be employed to assure that BP does not exceed 180/110 mmHg, in which case the protocol will be discontinued for safety.

*Sodium excretion* We will assess sodium excretion in urine samples collected over 24 h period as previously described by Rakova et al. [19]. Briefly, a 24-h urine will be collected by participants in aliquots, transported to the laboratory and sodium measured. An average sodium excretion will be calculated and used as a surrogate of dietary intake.

##### Procedure for aim 2

Blood measurements will be conducted three times (Fig. 1) at baseline (day 7 before low salt intake is commenced), day 14 and on day 21 (last day of high salt intake). Heparinized blood specimens will be collected to compare IsoLGs, inflammatory markers (IL-17A, IL-6, TNF-α, IFN-γ) and immune activation markers (CD80, CD86, CD83) in all four groups. Daily BPs will be collected and a 24-h BP on the last day of each phase.

Laboratory procedures and flow cytometry analysis are elaborated in Additional file 1.

#### Statistical analysis

We will use FlowJo software (Tree Star, Inc.) for flow cytometry data analysis. STATA version 15 or SPSS version 22 and Graph pad prism version 8 will be used for statistical inferences and to determine if dietary salt is associated with inflammation and/or immune activation in HIV. Analysis of variance or the Kruskal–Wallis test to compare pro- and anti-inflammatory cytokines and cells, activation markers and hypertension status in all four groups will be employed followed by the Dunnett post hoc test for pairwise comparisons or the Tukey test to compare all the groups with the control (HIV negative normotensive) group. We will also compare the means of the markers of immune cell activation between salt-sensitive and salt-resistant subjects, dichotomized by a drop of ≥ 10 mmHg in SBP owing to salt loading, as described above. For this purpose, we will carry out unpaired t-tests for normally distributed data or Mann–Whitney tests for non-normally distributed data.

Regression models will be used to examine the relationship between outcome variables and selected determinant factors, to determine the impact of each variable on the outcomes and to control for confounding.

### Discussion

Murine model and few human studies have shown that excess dietary salt induces BP elevation and activates cells of the innate and adaptive immune system [14, 20]. The interaction between dietary salt, BP and immune activation is a fairly new concept with recent evidence that salt can accumulate in skin without commensurate water retention and induce inflammation [1]. Furthermore, high consumption of dietary salt correlates positively with BP and is associated with the development or exacerbation of hypertension [21–23].

**Fig. 2:**
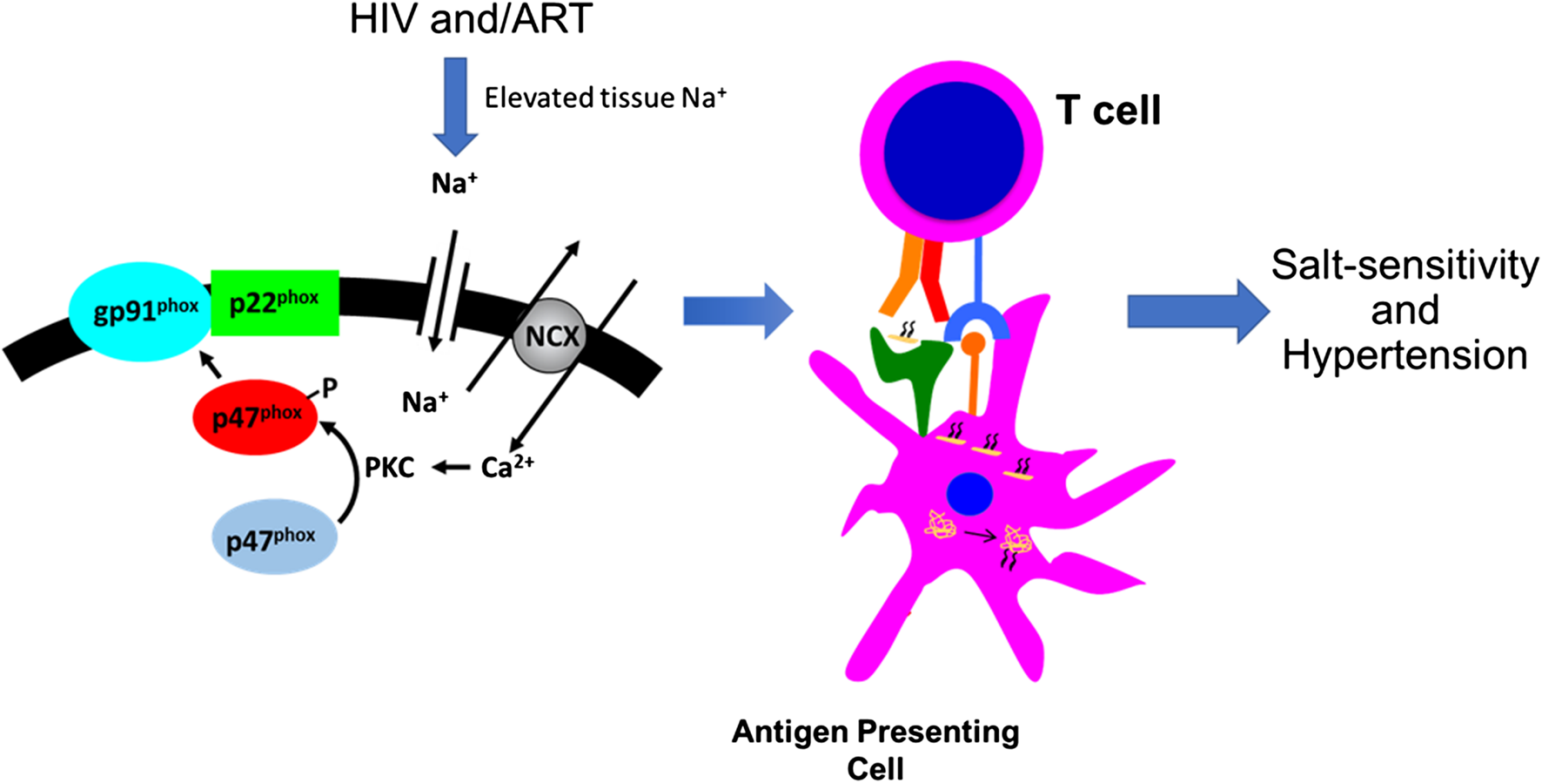
HIV and Salt-sensitive hypertension working Hypothesis. We hypothesize that HIV infection and treatment can lead to increased accumulation of sodium in tissues. This can activate the immune system leading to hypertension. Sodium enters antigen presenting cells and is exchanged for calcium through the sodium hydrogen exchanger 1. Calcium activates protein kinase C (PKC), which then activates NADPH oxidase by phosphorylating its subunit p47^phox^. This leads to increased production of superoxide with subsequent formation of immunogenic isolevuglandin (IsoLG)-protein adducts. IsoLGs activate DCs which in turn promote T cell proliferation and production of cytokines that contribute to salt-sensitive hypertension [4]

Previous studies have found that dendritic cells (DCs) accumulate isolevuglandin (IsoLG)-protein adducts during hypertension [4]. IsoLGs are highly reactive products of oxidation of fatty acids that rapidly adduct to lysines on proteins and their accumulation is associated with DC activation [4]. Recent studies have established that elevated Na+ is a potent stimulus for IsoLG-protein adduct formation in murine DCs [20]. Na+ enters DCs through amiloride sensitive transporters. Intracellular Na+ is exchanged for calcium (Ca2+) via the Na+/Ca2+ exchanger. Ca2+ activates protein kinase C (PKC) which in turn phosphorylates the NADPH oxidase subunit p47phox. This leads to activation of the NADPH oxidase, increased superoxide (O2·–) and IsoLG-protein adduct formation [20]. Adoptive transfer of salt-exposed DCs primes hypertension in response to a sub-pressor dose of angiotensin II and IsoLG-protein adduct formation is absent in mice lacking the NADPH oxidase and pharmacological scavenging of IsoLGs prevents DC activation, hypertension and end-organ damage [4, 20] (Fig. 2).

These studies in mice suggest that the immune system can exhibit salt-sensitivity, however the role of Na+ in activating human antigen presenting cells such as monocytes, and the interplay between elevated Na+ and salt sensitivity, has not been defined in the context of HIV. Sodium intake is positively associated with BP and accounts for much of the age-related increase in BP [24, 25]. Independent of other factors, excess intake of salt is associated with an increased risk of stroke [26, 27], cardiovascular disease and other adverse outcomes including death [28]. However, the effects of salt on BP may vary among individuals due to salt-sensitivity and salt resistance [26, 29]. This study will identify patients with or without salt-sensitive hypertension among HIV-negative and HIV-positive participants and examine levels of IsoLG-protein adducts and the activation markers of monocytes.

There are no studies known to us that have studied salt-sensitive hypertension and immune activation in PLWH. With this novel perspective, it is expected that PLWH may exhibit increased salt-sensitivity and immune activation compared to HIV negative individuals. This study has the potential to create points of intervention that will improve the management of hypertension and prevent dietary salt-related hypertension in future studies. Moreover, there is evidence that in the general population of Zambia, salt consumption exceeds that recommended by WHO [22]. This study is therefore, of clinical interest.

#### Strengths of the study

1. This is likely, the first study known to us that will explain or report a possible relationship between HIV, immune activation and salt-sensitive hypertension.
2. Generally, salt consumption in Africa and Zambia is higher than recommended. With increasing incidence of hypertension, knowledge about a possible interaction in this research is of clinical interest.

## Limitations

- Sodium excretion exhibits circaseptan rhythm and infra-radian rhythm depending on salt intake, increases or decreases in intake affects BP, However, a 24-h urine recovers about 95% of dietary salt intake.
- Though there are no established criteria for salt-sensitivity, we employ already published methods with maximal controls to avoid possible confounders. Information generated will provide baseline data for hypotheses generation for future planned studies.
- The underlying factors associated with HIV, immune activation and salt-sensitive hypertension are multifactorial, many factors not studied are likely to confound the results of the study. However, the experimental nature of the study will minimize confounding.
- This proposed study cannot determine causality and exhaust the mechanism and possible interactions between salt, immune system and HIV. Hence, further studies are warrantable.

## Additional file

**Additional file 1.** Laboratory procedures.

## Data Availability

All data generated or analyzed during this study are included in this published article. For other data, these may be requested through the corresponding author.

## Abbreviations

AMBP: ambulatory blood pressure
ART: antiretroviral therapy
BMI: body mass index
BP: blood pressure
CRP: C-reactive protein
DC: dendritic cell
DMSO: dimethyl sulfoxide
ECG: electrocardiogram
GM-CSF: granulocyte macrophage stimulating factor
HISH: HIV, Immune-activation and salt-sensitive hypertension study
FMO: flow minus one
HEPES: 4-(2-hydroxyethyl)-1-piperazineethanesulfonic acid
HIV: human immunodeficiency virus
IFN-γ: interferon gamma
IL: interleukin
IsoLG: isolevuglandins
LCH: Livingstone Central Hospital
MIFlowCyt: Minimum Information about a Flow Cytometry Experiment
PBMC: peripheral blood mononuclear cells
PLWH: people living with HIV
PMA: phorbol-12-myristate 13-acetate
PKC: protein kinase C
RPMI: Roswell Park Memorial Institute
sCD14: soluble CD14
TNF-α: tumor necrosis factor alpha
UVP: University of Zambia Vanderbilt partnership
WB: whole blood
WC: waist circumference
WHO: World Health Organizations
WHR: waist-to-hip ratio
