# Supplementary material for: HIV, immune activation and salt-sensitive hypertension (HISH): a research proposal
Source: BMC Res Notes. 2019 Jul 16;12:424. doi: 10.1186/s13104-019-4470-2 (PMC6636142; doi:10.1186/s13104-019-4470-2)
Supplement: Supplementary file 1 — Additional file 1. Laboratory procedures. [file 13104_2019_4470_MOESM1_ESM.docx]

**Additional file 1. Laboratory procedures**

**Laboratory Procedures**

*Whole blood assays:*

Unstimulated: 4*ml* of heparinized whole blood (WB) will be drawn and flow cytometry performed for markers of activation. Inflammatory cytokines will be measured in plasma.

Stimulated: heparinized whole blood (WB) will be diluted in a 1:1 ratio in Roswell Park Memorial Institute (RPMI) 1640 medium containing 25mM 4-(2-hydroxyethyl)-1-piperazineethanesulfonic acid (HEPES), and L-glutamine (Thermo Fisher Scientific). A cell activation cocktail (Biolegend USA) containing 40.5 µM of phorbol-12-myristate 13-acetate (PMA), ionomycin (669.3 µM), and Brefeldin A (2.5 mg/ml) in dimethyl sulfoxide (DMSO) (500X) will be added to each mL of cell suspension and incubated for 4-6 hours in 5% CO_2_ at 37°C. Following this, red blood cell lysis buffer will be added and incubated for 5 minutes at room temperature. The samples will then be centrifuged and the supernatant stored for cytokine measurement. A Cell Staining Buffer (Biolegend USA) will then be added and mixed with the cell pellet, then centrifuged and supernatant discarded and cell surface immunofluorescent staining performed.

*In-vitro experiments*

Stimulating cells with salt, in vitro, to measure salt-sensitivity will also be conducted to answer whether cells coming from salt-sensitive people are also salt-sensitive in vitro. Total PBMCs isolated from salt-sensitive and salt resistant individuals will be exposed to normal salt (150mM NaCL) or high salt (190mM NaCl) for 48 hours and then evaluate for the effect of low and high salt on cell subtypes by flow-cytometry. Cytokines secreted in media will also be measured.

*Intracellular staining*

Fixation, permeabilization and intracellular staining will be performed using standard protocols. Briefly, Cells will be fixed using a fixation buffer (Biolegend USA) and re-suspended in intracellular staining perm wash buffer (Biolegend USA) and then fluorophore-conjugated antibodies of interest added. After incubation cells will be analyzed with appropriate controls.

*Peripheral blood mononuclear cell (PBMCs) assays*: Heparinized blood samples (40 ml) will be obtained and PBMCs isolated by Ficoll-gradient. Monocytes and lymphocytes will be isolated from the PBMC by magnetic labeling and negative selection using the Miltenyi isolation kits. Cells will be activated as described above and analyzed using flow cytometry. T cells will be incubated for 72 hours supplemented with CD28 in RPMI medium.

*Other tests:* Lipid profile, kidney and liver function tests will also be conducted on collected blood samples. Urine samples will be collected for urine analysis microscopy, creatinine, protein and other analytes obtained by dip stick strips. Participants will also undergo electrocardiogram investigations.

*Flow cytometry:*

Standard manufacturer procedures will be followed using the FACS Calibur (Becton Dickinson USA). We will identify monocytes as CD45+/CD14+ cells and will further examine intermediate (CD14^++^/CD16^+^) and non-classical monocytes (CD14^-^/CD16^+^). A general characterization of mononuclear cells by flow cytometry will be performed for other inflammatory cells including total leukocytes (CD45+ cells), B cells (CD45+/CD19+), total T cells (CD45+/CD3+ cells), and the T cell subtype (CD3+/CD8+ and CD4+) cells. Expression of markers of activation CD80, CD83, and CD86 on monocytes and T lymphocytes will be performed. The LIVE/DEAD™ Fixable Violet Dead Cell Stain Kit (Thermo Fisher L34955) will be employed to determine the viability of cells. For each experiment, we gate on single live cells and use flow minus one (FMO) controls for each fluorophore to establish the gates.

For reporting of flow cytometry data, we will use the international standard guidelines called Minimum Information about a Flow Cytometry Experiment (MIFlowCyt) v. 1.0.
